# Challenges in Caring for People with Cardiovascular Disease through and beyond the COVID-19 Pandemic: The Advantages of Universal Access to Home Telemonitoring

**DOI:** 10.3390/healthcare11121727

**Published:** 2023-06-12

**Authors:** Luminita Iliuță, Andreea Gabriella Andronesi, Marius Rac-Albu, Florentina Ligia Furtunescu, Mădălina-Elena Rac-Albu, Alexandru Scafa-Udriște, Horațiu Moldovan, Eugenia Panaitescu

**Affiliations:** 1 Medical Informatics and Biostatistics Department, University of Medicine and Pharmacy “Carol Davila”, 050474 Bucharest, Romania; 2 Cardioclass Clinic for Cardiovascular Disease, 031125 Bucharest, Romania; 3 Nephrology Department, University of Medicine and Pharmacy “Carol Davila”, 050474 Bucharest, Romania; 4 Nephrology Department, Fundeni Clinical Institute, 022328 Bucharest, Romania; 5 Department of Public Health and Management, Faculty of Medicine, University of Medicine and Pharmacy “Carol Davila”, 050474 Bucharest, Romania; 6 Department of Cardio-Thoracic Pathology, University of Medicine and Pharmacy “Carol Davila”, 050474 Bucharest, Romania; 7 Department of Cardiology, Clinical Emergency Hospital, 014461 Bucharest, Romania; 8 Department of Cardiovascular Surgery, Clinical Emergency Hospital, 014461 Bucharest, Romania; 9 Academy of Romanian Scientist (AOSR), 3 Ilfov Street, 050044 Bucharest, Romania

**Keywords:** telemedicine, cardiovascular prevention, telemedicine, coronary artery bypass grafting, remote monitoring, coronary angioplasty, cardiovascular risk, cholesterol, COVID-19

## Abstract

(1) Background: Cardiovascular prevention was left in second place during the COVID-19 pandemic and the use of telemedicine turned out to be very useful. We aimed to evaluate the effectiveness of a telemedicine application for remote monitoring and treatment adjustments in terms of improving cardiovascular prevention. (2) Methods: A prospective study of 3439 patients evaluated between the 1st of March 2019 and the 1st of March 2022, in the pre-pandemic period by face-to-face visits and during the pandemic by teleconsultations or hybrid follow-up. We compared four periods: pre-pandemic—Pre-P (1 March 2019–1 March 2020), lockdown—Lock (1 March–1 September 2020), restrictive-pandemic—Restr-P (1 September 2020–1 March 2021), and relaxed–pandemic—Rel-P (1 March 2021–1 March 2022). (3) Results: The average values of total cholesterol (TC), LDL cholesterol, triglycerides, uric acid, and glucose had an increasing trend during Lock and Restr-P, and they decreased close to the baseline level during the Rel-P, with the exception of glucose which remained elevated in Rel-P. The number of patients with newly discovered DM increased significantly in the Rel-P, and 79.5% of them had mild/moderate forms of COVID-19. During Lock and Res-P, the percentage of obese, smoking, or hypertensive patients increased, but probably through the use of telemedicine, we managed to reduce it, although it remained slightly higher than the pre-pandemic level. Physical activity decreased in the first year of the pandemic, but in Rel-P people became more active than before the pandemic. (4) Conclusions: The use of telemedicine for cardiovascular prevention seems to yield favorable results, especially for secondary prevention in the very high-risk group and during the second year.

## 1. Introduction

The unprecedented global COVID-19 pandemic had a significant impact on health services in terms of patient care but especially negatively influenced primary and secondary prevention. There was a significant reduction in the access to health services imposed by the adopted restrictions and reallocation of resources and priorities to caring for patients with COVID-19 and preventing the spread of the virus, thus causing a significant reduction in consultations and so-called non-life-threatening procedures. As the pandemic took hold, the importance of cardiovascular prevention was overshadowed, the focus of all cardiologists moving more toward reaction, intervention, and treatment and away from prevention, motivation, and counseling [1,2,3,4,5]. 

In Romania, a state of national emergency was declared from 1 March to 16 May 2020 and imposed isolation, which resulted in medical countermeasures, including the cancelation of non-urgent medical and surgical activities, to preserve intensive care capacity and limit viral spread between hospitals. Patients with cardiovascular diseases are a particularly vulnerable population [2]. They may decompensate or require hospitalization due to increased susceptibility to infection, but also due to reduced physical activity related to isolation, withdrawal from psychosocial support networks, and difficulties in providing medical assistance. On the other hand, cardiovascular diseases and cardiovascular risk factors, such as obesity, diabetes, and hypertension, have been identified since the beginning of the pandemic both as frequent comorbidities among patients who required hospitalization for COVID-19 and also as markers for more severe forms of illness and death.

In our country, the public health crisis triggered by the COVID-19 pandemic dramatically changed preventive cardiology services, both primary and secondary, especially in the first period, as well as the provision of measures to implement a healthy lifestyle. These are fields with long-term benefits and, in the context of a landscape full of immediate challenges, they seemed counterintuitive and, consequently, they were marginalized. As social distancing restrictions were extended for months and had important implications for cardiovascular health, innovative efforts were needed to adapt current approaches to CVD prevention [3]. However, efforts were made in all countries to prevent cardiovascular diseases because, although COVID-19 represented the most imminent health emergency, cardiovascular diseases remain the leading cause of death worldwide [4,5]. Likewise, the recommendations in Australia and New Zealand aimed at increasing the use of a range of electronic health platforms, with the integration of research programs to evaluate their utility and thus improve secondary prevention beyond the pandemic [4]. In the review published by Duffy et al., they sought to highlight what the pandemic has taught us about caring for the vulnerable patients who were most affected—older adults and those facing adverse socioeconomic circumstances—and who continue to be affected by cardiovascular diseases (CVDs). They also identified opportunities for innovative ways to prevent CVD, spurred by the overnight adoption of telemedicine and other remote cardiac care models [6]. In another study published by Dale et al., they analyzed the number and percentage changes of drugs dispensed for several CVDs (with an emphasis on arterial hypertension, hypercholesterolemia, and diabetes). They used data that included 1.32 billion records of CVD medicines dispensed in England, Scotland, and Wales between April 2018 and July 2021. They observed a fall in the dispensing of CVD medicines between March 2020 and July 2021, with the number of people initiating treatment significantly reduced (by 491, 306). They estimated that this decrease could result in 13,662 additional cardiovascular events, including 2281 cases of myocardial infarction and 3474 cases of stroke, if people remained untreated over their lifetime. Taking these results into account, additional primary and secondary prevention measures are needed [7]. 

On the other hand, looking back, the pandemic also had positive aspects as it helped us to find alternative methods. Telemedicine is a useful tool to provide support and care to stable patients, reserving direct patient–provider contact for emergent/urgent situations [8]. During the pandemic, new applications and platforms for doctor–patient interaction were developed, thus providing a unique opportunity to improve preventive care [9,10,11]. In this context, we immediately adapted our dedicated application in order to maintain follow-up with stable patients with the minimum physical contact possible and we reserved the in-person evaluations and interventions for urgent situations or unstable patients. 

Some previous studies evaluated the relationship between the cardiovascular risk score and COVID-19 and the impact of high cholesterol levels on the evolution and prognosis of patients with COVID-19 [2]. However, there are no published studies regarding the evolution of cholesterol levels and the cardiovascular risk score during the COVID-19 pandemic in patients with various levels of cardiovascular risk.

That is why, the first aim of our study was to analyze how the main cardiovascular risk factors evolved during the COVID-19 pandemic in the months of quarantine, isolation, and physical inactivity in patients from a cardiovascular clinic in Bucharest, depending on the patient’s risk level.

We also analyzed the usefulness of the dedicated telemedicine application for remote multiparametric monitoring of patients, adjustment of therapeutic regimens, and promotion of a healthy lifestyle in terms of improving remote cardiovascular prevention.

## 2. Materials and Methods

### 2.1. Study Population, Setting, and Data Collection

We carried out a prospective study in 3439 patients recorded in the Cardioclass Clinic for cardiovascular diseases, evaluated between the 1 March 2019 and the 1 March 2022. Patients were eligible for enrolment in the remote monitoring program if they had been evaluated in our clinic and registered in the dedicated application within the preceding 12 months before the beginning of the lockdown. The study protocol was approved by the Ethics Committee of the Cardioclass Clinic for Cardiovascular Diseases, through Decision no. 305/07 January 2019. All patients included in this study were informed about the study’s purpose and signed the informed consent form authorizing prospective data collection for research purposes. The form of the informed consent is part of the study protocol.

A total of 4643 patients registered in the Cardioclass application data who signed the informed consent before 1 February 2019 were eligible for the study. Finally, after applying the exclusion criteria, or due to the fact that they were absent from the follow-up visits, the studied group included 3439 patients (Supplementary Figure S1).

In the pre-pandemic period (from 1 March 2019 to 1 March 2020), the standard follow-up of the patients consisted of in-person appointments (a minimum of one appointment per trimester for patients with a high or very high risk and a minimum of one appointment per year for patients with a medium or low risk) with a physician consultation, electrocardiogram, echocardiographic examination, and/or ambulatory electrocardiogram (EKG) or blood pressure monitoring. All patients also had access to a specific phone number and dedicated email. We provided learning instruments to instruct the patients to follow important parameters, these being evaluated by the attending physician at each visit. Follow-up phone calls were scheduled to check patients’ symptoms and to eventually adjust drugs, mainly for dyslipidemia, arterial hypertension, or antianginal medication (nitrates). Whenever necessary, according to blood pressure evolution, angina symptoms, or a poor response to oral nitrates, patients were admitted to our clinic for re-evaluation or to have intravenous diuretics or nitrates (guided by a pre-specified protocol established by the clinic doctors based on international guidelines regarding the doses of diuretics, nitrates, or anti-arrhythmics, depending on the specific clinical situation).

During the lockdown and the restricted period (from 1 March 2020 to 1 March 2021), the in-person appointments were drastically reduced, being limited to urgent situations. All the pre-scheduled appointments were converted to teleconsultations in order to identify which patients would need in-person care. In order to check drug adherence, apart from the phone inquiry by our dedicated team, we monitored the need for drug prescription renewal. Many blood tests were made in local laboratories and home-based phlebotomy was allowed. All the stress tests had to be canceled (including the exercise stress test and stress echocardiography) due to COVID-19 constraints.

During the pandemic, all patients were monitored with a multiparametric application that incorporated symptoms, blood pressure (BP), heart rate (HR), blood test results, and electrocardiograms (via Istel HR-2000 6-lead ECG Recorder with four built-in electrodes as remote monitoring system—Diagnosis SA, Bialystok, Poland). The telemedicine application allowed weekly transmissions of blood pressure, heart rate, and responses to questions relating to five coronary artery disease (CAD) symptoms to a remote monitoring server. We collected responses monthly to stay up-to-date on key cardiovascular risk factors and track the changes in the cardiovascular risk score. All collected data were monitored by a specialized team in the remote monitoring application. The monitored parameters were subject to 10 programmable alerts based on critical absolute values or changes over time.

The main alerts for angina decompensation diagnosis were:

- Chest pain, chest tightness, or angina more than four times/day;
- Nitrate (nitroglycerin tablets) administration for chest pain, chest tightness, or angina more than four times/day;
- Limitation due to chest pain, chest tightness, or angina in dressing or walking indoors on level ground;
- Mean HR of more than 100 b/min for three consecutive days or paroxysmal atrial fibrillation.

In order to simplify the monitoring process and the alert system, the responses to questions related to angina symptoms were classified as Good, Attention, and Alert, using an adapted Seattle Questionnaire [12], as follows:

Good—No chest pain at rest and during daily activities; rarely chest pain at the most strenuous level of activity.

- No nitroglycerin for chest pain, chest tightness, or angina since the last visit;
- Non-restricted daily activities;
- No limitation in the enjoyment of life due to chest pain;
- No depression, you have a zest for life.

Attention—Chest pain, chest tightness, or angina three or more times per week but not every day.

- You take nitroglycerin for chest pain, chest tightness, or angina three or more times per week but not every day;
- Moderate limitation of daily activities due to chest pain (limitation in running or jogging, lifting or moving heavy objects (e.g., furniture, children), participating in strenuous sports (e.g., swimming, tennis);
- Slight limitation in the enjoyment of life due to chest pain;
- You feel more depressed than usual.

Alarm—You have pain, pressure, or tightness in your chest more than one time/day.

- You take nitroglycerin for chest pain, chest tightness, or angina daily;
- Severe limitation of daily activities due to chest pain (limitations in dressing yourself, walking indoors on level ground, or showering);
- Severe limitation in the enjoyment of life due to chest pain;
- You feel very depressed, you often think that you may have a heart attack or die suddenly.

The cardiovascular risk score was calculated upon enrollment in the study, based on each patient’s record from our dedicated application, as a sum of risk factors (older age, male, family history of cardiovascular disease, arterial hypertension, high levels for low-density lipoprotein cholesterol—LDL or triglycerides, diabetes, smoking or secondhand smoke exposure, obesity, unhealthy diet, physical inactivity, and stress). At each visit, an updated (corrected) cardiovascular risk score was calculated as the sum of non-modifiable risk factors (older age, male, and family history of cardiovascular disease) and modifiable risk factors. For calculation of the corrected cardiovascular risk score, we took into account a modifiable risk factor only when it was uncontrolled, either by changing life habits or by drug treatment (uncontrolled arterial hypertension, high level of LDL cholesterol or triglycerides corresponding to risk group, uncontrolled diabetes, smoking or passive smoking, obesity, unhealthy diet, physical inactivity, and stress). 

We used the risk score calculated by our dedicated application for a more detailed analysis, separated by risk factors and the easier follow-up of patients, because it takes into account more risk factors than the classic scores used. However, to divide the patients into risk groups, we calculated their SCORE2 risk prediction with a new algorithm that has been used to estimate the 10-year risk of cardiovascular disease in Europe [13]. The maximum cardiovascular risk score calculated by our application was 10 and it had a good correlation with the SCORE2 model of the European Society of Cardiology from 2021 (Pearson r = 0.72, *p* < 0.05). 

In the relaxed-pandemic (Rel-P) period (from 1 March 2021 to 1 March 2022), we provided a hybrid follow-up of the patients with telemedicine consultation using our dedicated multiparametric application and in-person appointments (minimum of one appointment per year) with a physician consultation, electrocardiogram, echocardiographic examination, and/or ambulatory EKG or blood pressure monitoring.

### 2.2. Study Periods and Variables of Interest

Variables of interest were compared between four periods: pre-pandemic—Pre-P (1 March 2019–1 March 2020), lockdown—Lock (1 March–1 September 2020), restrictive-pandemic—Restr-P (1 September 2020–1 March 2021), and relaxed–pandemic—Rel-P (1 March 2021–1 March 2022). 

Blood tests, physiological variables, and symptoms were noted at the enrollment and during monitored periods for three years. The remote monitoring application was used to collect data on blood pressure, heart rate, EKG, symptoms, blood tests, alerts, angina decompensation events, medication changes, consultations, and details of hospitalizations. Angina decompensations were reported by the cardiologists from the remote monitoring center and resulted in an increase in antianginal treatment.

All patients with CAD received the standard treatment for this disease with lipid-lowering agents, beta-blockers, angiotensin-converting enzyme inhibitors, antiplatelets, nitrates, and anticoagulants when indicated. In addition, we recorded in the dedicated application the COVID-19 illnesses and their form (mild, medium, or severe) as well as the anti-COVID-19 vaccination (date, type of vaccine, and the number of doses).

### 2.3. Statistical Analysis

Quantitative data were reported as the mean ± standard deviation (SD) or as the median with the 25th and 75th percentile, whereas qualitative data were summarized as absolute values with the corresponding percentages. Parametric or nonparametric paired tests were used to compare two time point estimates (paired Student *t*-test or Wilcoxon-rank test). A repeated-measures analysis of variance (ANOVA) was used to compare variables assessed at three different time points after checking for normality and homoscedasticity (homogeneity of variances) with the conventional tests. When these assumptions were violated, the non-parametric repeated ANOVA (Friedman test) was used. When the F-ratio of the ANOVA or the Friedman test reached a critical level (corresponding to a *p* < 0.05), a post hoc analysis with *p*-value adjustment for multiple comparisons was used. Categorical paired nominal data at two time points were compared with the McNemar test.

Furthermore, we used ANOVA RM (repeated measures) to compare the parameters measured at the four time points. In addition, to confirm the hypothesis that time affected the averages of the measured parameters (which was the first aim of our study), we also used linear mixed models.

We used the Pearson Chi-Square to analyze if there was a statistically significant difference in prevalence (binary outcome) between the 3+ groups. Parametric or nonparametric paired tests were used to compare two time point estimates (paired Student *t*-test or Wilcoxon-rank test) and the baseline characteristics of the study groups (Chi-Square and ANOVA). 

In order to check the conditions of the models, the values for asymmetry and kurtosis between −2 and +2 were considered acceptable in order to prove normal univariate distribution [14,15]. Additionally, we considered a normal distribution when skewness was between −2 and +2 and kurtosis was between −7 and +7 [14]. Mauchly’s test of sphericity was significant (*p* < 0.001), so the assumption of sphericity was not met. We used the Greenhouse–Geisser correction when Epsilon < 0.75 and the Huynh–Feldt correction when Epsilon > 0.75. After three time points, the *p*-values were adjusted for multiple comparisons. All tests were performed two-sided and a *p*-value < 0.05 was considered statistically significant. Statistical analysis was performed using SPSS version 23.0.

## 3. Results

According to 2019 ESC/EAS Dyslipidemia guidelines [15], and depending on their risk group, patients were divided into the following three groups:

1. Group 1—Very high risk—194 patients with documented atherosclerotic cardiovascular disease (ASCVD), coronary artery disease (CAD), or diabetes mellitus (DM) with target organ damage, ≥3 major risk factors or early onset of type 1 DM of long duration (>20 years), severe chronic kidney disease (eGFR < 30 mL/min/1.73 m^2^), and calculated SCORE ≥ 10% for 10-year risk of fatal CVD;
2. Group 2—High risk—1611 patients with markedly elevated single risk factors (in particular TC > 310 mg/dL, LDL cholesterol > 190 mg/dL or blood pressure ≥ 180/110 mmHg) or patients with DM without target organ damage, with DM duration ≥ 10 years or another additional risk factors, or a calculated SCORE between 5% and 10% for 10-year risk of fatal CVD;
3. Group 3—Medium and low risk—1634 patients who were not in the high or very high-risk groups.

Information regarding the demographic, clinical characteristics, and treatment of the patients was obtained in the last appointment before the pandemic period and is presented in Table 1. 

The majority of patients received target doses of medication in accordance with the current guidelines. At the moment of enrollment, the patients received treatment for dyslipidemia, as is shown in Table 2.

The percentage of anti-COVID-19 vaccinated patients in the whole study group was 78.89% (2713 pts), and by risk groups, it was 97.42% (189 pts) for the very high-risk group, 87.95% (1417 pts) for the high-risk group and 68.71% (1107pts) for the medium and low-risk group (*p* = 0.000001, Likelihood Ratio).

**Figure 1:**
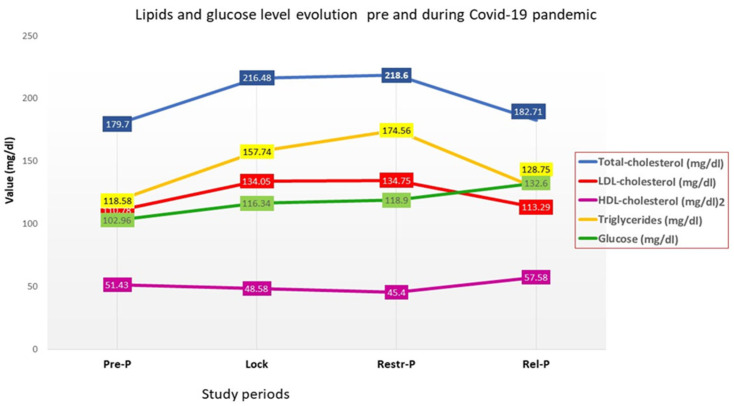
Lipids and glucose level evolution before and during the COVID-19 pandemic.

During the pandemic, the average values of TC, LDL—low-density lipoprotein—cholesterol, triglycerides, and glucose had an increasing trend during Lock and Restr-P compared to Pre-P, with statistically significant differences. During the Rel-P period, lipidogram parameters decreased close to the baseline level, regardless of the risk group (Figure 1).

The average blood glucose level in the studied group had the same increasing trend during Lock and Restr-P but remained elevated in Rel-P, probably due to the fact that many of the patients developed diabetes during the pandemic (Figure 1). The division into risk groups was decided when enrolling in the study. Some of the patients who were in the medium and low-risk groups at the beginning of the pandemic moved into the high- or very high-risk groups at the end of the pandemic.

The HDL—high-density lipoprotein—cholesterol mean value decreased during Lock and Restr-P compared to Pre-P, but, during Rel-P, reached a higher value than before the pandemic. In addition, the uric acid mean values showed the same increasing trend during Lock (6.10 ± 2.696 mg/dL) and Restr-P (6.15 ± 1.790 mg/dL) compared to Pre-P (5.20 ± 3.768 mg/dL) and decreased during Rel-P (5.75 ± 2.755 mg/dL) but it remained higher than pre-pandemic.

Regarding the cardiovascular risk score and the other modifiable cardiovascular risk factors recorded in the telemedicine application, they also had an increasing trend during the pandemic, as illustrated in Table 3. The highest growth trend was recorded in the number of patients diagnosed with diabetes, which increased significantly throughout the pandemic. In addition, medium BMI increased during the pandemic and the percentage of obese persons increased significantly during Lock. In terms of physical activity, the percentage of inactive people increased during the pandemic, but at the end of the pandemic, it decreased significantly compared to the pre-pandemic period.

For patients with known coronary disease, the application also recorded a remote electrocardiogram once a week or in case of worsening symptoms. Although most of the patients from the very high and high-risk groups reported HR, those in the medium-risk group with normal BP and HR did not have the monitoring tools for these parameters and, consequently, the information obtained from them was less.

Participants recorded blood tests for the four time periods: Pre-P, during Lock, Restr-P, and Rel-P. Because of missing data in some periods and in order to be able to draw conclusions based on a more robust statistical analysis, we used the Paired Samples T Test (which considers only the number of pairs with values at the two time points). Normality checks were carried out on the residuals and original values. The normality tests showed a significant difference, but the values for skewness and kurtosis were within acceptable limits. The large number of values used (more than 1000 for each test) assured us that the method is robust even if the normality conditions were not met. 

So, a comparison between the four analyzed periods (Pre-P with Lock, Restr-P, and Rel-P, Lock with Restr-P and Rel-P, and Restr-P with Rel-P) using the Paired Samples T Test showed a significant increase in total cholesterol, LDL cholesterol and triglycerides (TGL) level during Lock and Restr-P compared to Pre-P, irrespective of the risk group. 

A repeated measures ANOVA with a Greenhouse–Geisser correction showed that the mean TC differed significantly between time points [F(1.878, 2292.783) = 1216.637, *p* < 0.001]. Post hoc tests using the Bonferroni correction revealed that TC increased compared to Pre-P by an average of 35.130 mg/dL after 1 year during Lock, by 41.123 mg/dL during Restr-P, and dropped to 8.937 mg/dL after 3 years, compared to the baseline (Table S1). In addition, TC increased by 5.993 mg/dL between Lock and Restr-P and decreased by 44.067 mg/dL between Lock and Rel-P and by 50.060 mg/dL between Restr-P and Rel-P. There was a significant difference between each pair of time points (*p* = 0.000001).

In addition, the mean LDL cholesterol values differed significantly between time points {F(2.038, 2349.588) = 846.944, *p* = 0.000001}. Post hoc tests using the Bonferroni correction revealed that LDL cholesterol increased compared to Pre-P by an average of 21.932 mg/dL after 1 year during Lock, by 24.670 mg/dL during Restr-P, and dropped by 5.001 mg/dL after 3 years compared to the baseline (Table S2). In addition, LDL cholesterol increased by 2.738 mg/dL between Lock and Restr-P. It decreased by 26.933 mg/dL between Lock and Rel-P and by 29.671 mg/dL between Restr-P and Rel-P. Moreover, we found a significant difference between each pair of time points (*p* = 0.000001).

For HDL cholesterol values analysis we used ANOVA repeated measures with a Huynh–Feldt correction. The mean HDL cholesterol differed significantly between time points {F(2.611, 2987.256) = 1195.152, *p* = 0.000001}. Post hoc tests using the Bonferroni correction revealed that HDL cholesterol decreased compared to Pre-P by an average of 4.627 mg/dL after 1 year during Lock, by 8.592 mg/dL during Restr-P, and increased by 6.113 mg/dL after 3 years compared to the baseline (Table S3). In addition, HDL cholesterol decreased by 3.966 mg/dL between Lock and Restr-P. It increased by 10.740 mg/dL between Lock and Rel-P and by 14.705 mg/dL between Restr-P and Rel-P (*p* = 0.000001).

For triglycerides (TGL), the mean values differed significantly between time points {F(2.496, 2975.483) = 929.321, *p* = 0.000001}, increasing compared to Pre-P by an average of 36.46 mg/dL after 1 year during Lock, by 52.62 mg/dL during Restr-P, and by 1.76 mg/dL after 3 years compared to the baseline (*p* = 0.000001) (Table S4). In addition, TGL increased by 17.540 mg/dL between Lock and Restr-P and decreased by 34.953 mg/dL between Lock and Rel-P and by 52.492 mg/dL between Restr-P and Rel-P.

In addition, the same statistical analysis revealed that the mean glucose differed significantly between time points {F(1.935, 2254.585) = 1501.401, *p* < 0.001}. Post hoc tests using the Bonferroni correction revealed that glucose increased compared to Pre-P by an average of 14.874 mg/dL after 1 year during Lock, by 19.940 mg/dL during Restr-P, and by 34.500 mg/dL after 3 years compared to the baseline (Table S5). In addition, glucose levels increased constantly with 5.066 mg/dL between Lock and Restr-P, 19.626 mg/dL between Lock and Rel-P, and 14.560 mg/dL between Restr-P and Rel-P. There was a significant difference between each pair of time points (*p* = 0.000001).

The blood glucose values, however, maintained an increasing trend throughout the pandemic (Figure 1), with the highest increase in the Pre-P–Rel-P period, followed by Pre-P–Restr-P, and Lock–Rel-P. During the Rel-P period, the number of patients with newly discovered DM increased significantly compared to previous periods and 79.5% of them had mild/moderate forms of COVID-19, as other studies showed [16,17].

The univariate regression analysis showed a statistically significant correlation between the mild or moderate form of COVID-19 infection and blood sugar levels > 110 mg/dL (r = 0.79 Pearson, *p* < 0.005).

In addition, the mean uric acid differed significantly between time points {F(1.277, 1294.526) = 76.555, *p* = 0.000001}, increasing by an average of 0.843 mg/dL after 1 year, 1.007 mg/dL after 2 years, and 0.352 mg/dL after 3 years compared to the baseline (*p* = 0.000001) (Table S6). In addition, uric acid increased constantly with 0.490 between Lock and Restr-P and 0.655 mg/dL between Lock and Rel-P, and decreased with 0.82 mg/dL between Restr-P and Rel-P.

**Figure 2:**
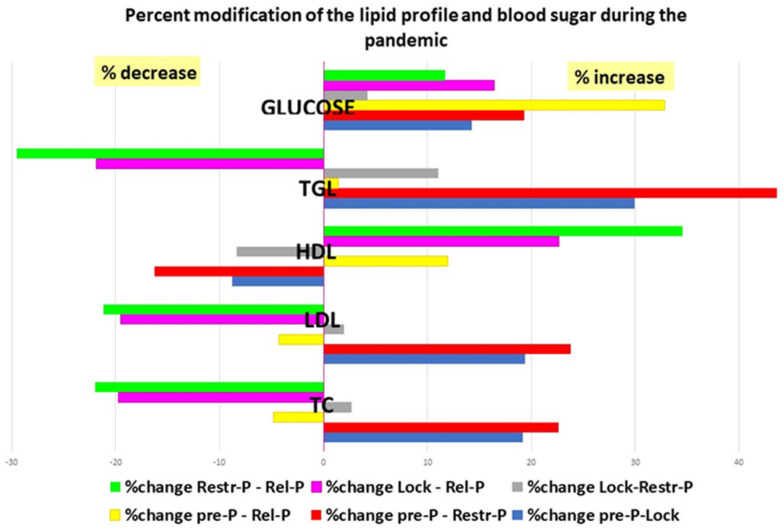
Percentage change in the lipid profile and glucose level during the pandemic. TGL—triglycerides; HDL—high-density lipoprotein; LDL—low-density lipoprotein; TC—total cholesterol; Pre-P—pre-pandemic; Restr-P—restrictive pandemic; Rel-P—relaxed pandemic; and Lock—lockdown.

The comparative analysis of the evolution of the TC, LDL, and TGL in the four study periods showed the highest increase between Pre-P and Restr-P, followed by Pre-P and Lock, and the greatest reduction between Restr-P and Rel-P, followed by Lock and Rel-P (Figure 2). Conversely, for HDL levels, the analysis showed a marked decrease in the first year of the pandemic with a maximum between Pre-P and Restr-P, followed by Pre-P and Lock, and an increase between Restr-P and Rel-P, followed by Lock and Rel-P.

Additionally, for all studied parameters, the results obtained using linear mixed models suggest that the time variable influenced the averages of the estimated parameters and that there were changes in means occurring over time (F-test).

Thus, for total cholesterol and LDL cholesterol, the mixed linear models showed that their averages for the Lock and Restr-P period were significantly increased compared to Pre-P. On the other hand, the average value for total cholesterol and LDL cholesterol in the Pre-P period was lower than in the Rel-P period, but this difference was at the limit of statistical significance (*p* = 0.053 resp. 0.072). For HDL cholesterol and blood sugar, all average values (from Pre-P, Lok, and Restr-P periods) were significantly lower compared to Rel-P. For TGL and uric acid, the mean values in the Lock and Restr-P periods were significantly increased compared to Rel-P, while their mean value was significantly lower in Pre-P compared to Rel-P.

In order to highlight more clearly how the main parameters of the lipidogram evolved according to the group of risk, we performed a differentiated statistical analysis by risk groups. This analysis also showed significant differences during the pandemic compared to the pre-pandemic period for all variables studied in all groups, but mainly in the high-risk group.

**Figure 3:**
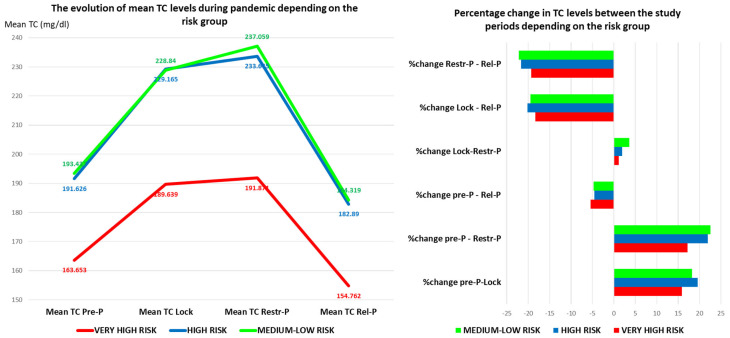
The evolution (value and percentage) of TC levels during the pandemic depending on the risk group. TC—total cholesterol; Pre-P—pre-pandemic; Restr-P—restrictive pandemic; Rel-P—relaxed pandemic; and Lock—lockdown.

Thus, the trend of variation in the average values of TC was similar in the three risk groups studied. The curve of the average values of TC in the high-risk group overlaps the curve corresponding to the medium-low risk group, which highlights the deficient primary prevention measures in this risk group. (Figure 3).

Regarding the percentage of variation in the TC values before and after the pandemic, it was higher in the medium-low or high-risk groups in the study intervals. The highest increase was recorded for the medium-low risk group in the Pre-P–Restr-P period, followed by the Pre-P–Lock period for the high-risk group, and the biggest reduction in the Restr-P–Rel-P period followed by the Lock–Rel-P period, regardless of the risk group. Between the lockdown period and the Restr-P period, there were no significant differences, only a slight increase in TC.

Compared to the beginning of the pandemic, the average level of TC during the Rel-P period decreased, especially in the very high-risk group, which may indicate adequate primary and secondary prevention measures by using the dedicated remote monitoring application.

Taking into account the main lipid marker from the guidelines, LDL cholesterol, the trend of variation in the average values of LDL cholesterol was similar in the three risk groups studied. The curve of the average values of LDL cholesterol in the high-risk group overlaps the curve corresponding to the medium-low risk group, which highlights the deficient primary prevention measures in this risk group.

**Figure 4:**
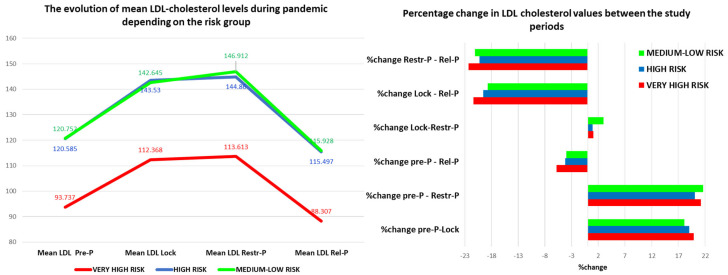
The evolution (value and percentage) of LDL cholesterol levels during the pandemic depending on the risk group. LDL—low density lipoprotein; Pre-P—pre-pandemic; Restr-P—restrictive pandemic; Rel-P—relaxed pandemic; and Lock—lockdown.

Regarding the percentage of variation in LDL cholesterol values before and after the pandemic, it was the highest in the very high-risk group in all study intervals. The highest increase was recorded in the Pre-P–Restr-P period, followed by the Pre-P–Lock period, and the biggest reduction in the Restr-P–Rel-P period followed by the Lock–Rel-P period, regardless of the risk group (Figure 4).

Between the Lock period and the Restr-P period, there were no significant differences, only a slight increase in LDL cholesterol. Compared to the beginning of the pandemic, the average level of LDL cholesterol during the Rel-P period decreased, especially in the very high-risk group, which may indicate adequate primary and secondary prevention measures by using the dedicated remote monitoring application.

**Figure 5:**
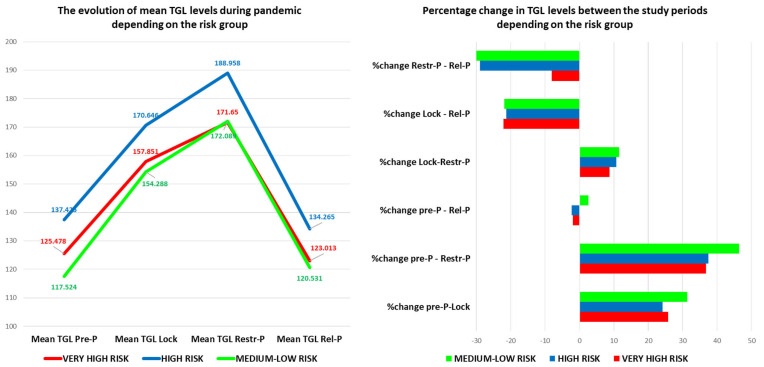
The evolution (value and percentage) of TGL levels during the pandemic depending on the risk group. TGL—triglycerides; Pre-P—pre-pandemic; Restr-P—restrictive pandemic; and Rel-P—relaxed pandemic.

The level of triglycerides followed the same trend but with the post-pandemic level slightly increased for the medium-low risk groups and a lower level than pre-pandemic for the very high- and high-risk groups (Figure 5). The highest percentage increase in the average TGL level was observed in the medium-low risk group for all analyzed periods, especially in the Pre-P–Restr-P interval. The reduction in the TGL level during Rel-P compared to Lock and Restr-P was observed in all risk groups.

Thus, the trend evolution of the lipid profile showed a significant increase in the Pre-Lock period, followed by a slight increase in the Lock–Restr-P period and a reduction in the Restr-P–Rel-P period, sometimes even to a lower level than before the pandemic for certain risk groups and certain parameters.

Compared to the pre-pandemic level, the average value of TGL for the high- and very high-risk groups, the average value of LDL cholesterol for all risk groups, and the average value of TC for all risk groups decreased.

**Figure 6:**
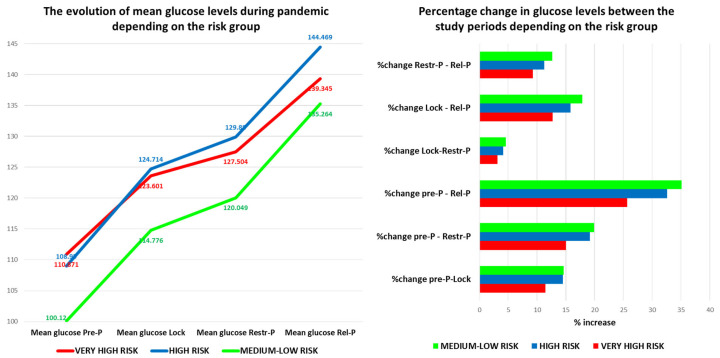
The evolution (value and percentage) of glucose levels during the pandemic depending on the risk group. Pre-P—pre-pandemic; Restr-P—restrictive pandemic; Rel-P—relaxed pandemic; and Lock—lockdown.

The blood glucose level registered a different evolution curve regardless of the risk group. The average blood sugar level had an increasing trend during the pandemic, with the highest average value being recorded in the high-risk group. Interestingly, the highest growth rate, regardless of the period, was recorded in the medium-low risk group, where the pre-pandemic average was low, followed by the high-risk group. The lowest percentage increase was in the very high-risk group, which is made up of patients with a history of cardiovascular disease and diabetes, in whom the secondary prevention measures seem to have been much more effective than the primary prevention measures for the other risk groups (Figure 6).

The blood glucose level increased in all risk groups, but especially in the medium-low risk group, probably also because the percentage of anti-COVID-19 vaccinated patients among the group was lower (68.71%), they had more frequent moderate–severe forms of COVID-19, and many of them acquired diabetes during the pandemic (6.3% of pre-pandemic diabetics, increased to 10.76% in Rel-P). Other studies also showed that COVID-19 is associated with aberrant glucose control, which can persist even after recovery. In our study, we found a good correlation between the infection with COVID-19 and newly discovered DM after the pandemic (Pearson r = 0.78, *p* < 0.005) [15].

The percentage of patients with hypertension, obesity, and who were smokers increased during the restricted pandemic, but by using the telemedicine application and an increase in medication we managed to reduce it, however, it remained higher than the pre-pandemic level (57.92% of patients with hypertension in Rel-P vs. 54.9% in Pre-P).

Physical activity decreased in the first year of the pandemic, but in Rel-P people became more active than before the pandemic.

In the pre-pandemic period, we noted a mean of 10 phone calls monthly with a dedicated nurse vs. a mean of 25 calls monthly during the pandemic. All of the pre-pandemic appointments, regarding clinical evaluation, were face-to-face and included echocardiographic evaluation. During the pandemic period, more than 80% of the appointments occurred online. The percentage of patients with in-person appointments significantly increased by the end of the pandemic period (March 2021–March 2022), compared to 2020–2021. We were able to successfully titrate antianginal, hypertension, and dyslipidemia medications even by online or phone call appointments with close monitoring of ambulatory blood pressure, heart rate, weight, symptoms, and blood test results, which were entered by the patients in the dedicated platform. Moreover, we did not record an increased rate of adverse drug events during the online follow-up.

In patients with arterial hypertension, during the two pandemic years (including the lockdown) the elevated blood pressure values were managed at home with an increase in antihypertensive drugs, taking into account the trends in systolic blood pressure (from the home monitoring system).

Regarding hospitalizations and emergency department (ED) visits due to angina decompensation, there was no statistically significant difference between the pre-pandemic and the pandemic period (angina hospitalizations—*p* = 0.73; ED visits *p* = 0.37). Furthermore, during the pre-pandemic period, there were 9 patients hospitalized and 19 patients treated in the ED due to angina exacerbation, compared with 8 patients hospitalized and 7 patients treated in the ED during the pandemic period.

The percentage of anti-COVID-19 vaccinated patients in the whole study group was 78.89% (2713 pts), and by risk groups, it was 97.42% (189 pts) for the very high-risk group, 87.95% (1417 pts) for the high-risk group, and 68.71% (1107 pts) for the medium-low risk group (*p* = 0.000001 Likelihood Ratio).

During the pandemic period, there were 297 patients diagnosed with COVID-19 (8.65%), 193 patients with a mild form and favorable outcome (no cardiovascular or respiratory complications, patients with only mild symptoms, 71.9% vaccinated), 84 patients with a moderate form requiring hospitalization (of which 65.5% were vaccinated), and 21 patients with severe disease, 2 of whom died (of which only 23.8% were vaccinated).

The finding that the telemedicine application was effective and safe in terms of controlling blood pressure values, cholesterol, and secondary prevention in general, as an approach based on clinical practice, is reassuring. However, the mean BP in Rel-P in the low-medium risk group was above the target limit, suggesting that there are also other factors involved, including clinical inertia and patient preference/adherence to primary prevention measures. Blood pressure and cholesterol control via telemedicine may be a better option for some people but not for others, resulting in effective heterogeneity between study groups.

Overall, the clinical condition of CAD patients under remote multiparametric monitoring was minimally affected by the lockdown restrictions, despite a marked decrease in conventional healthcare measures. This strategy, combined with measures to educate patients and increase their adherence, can mitigate the health risks associated with any pandemic.

## 4. Discussion

The COVID-19 pandemic significantly disrupted all cardiovascular disease prevention strategies. The related restrictions with the limitation of travel and physical contact primarily determined the reduction or cancellation of visits to the doctor, for all categories of patients, as well as delayed prevention. In addition, by working from home, the pandemic brought with it a reduction in physical activity and an increase in food intake and snacks, followed by weight gain. Social isolation was accompanied by mental stress, depression, and loneliness, as well as an increase in the time spent in front of the television and the favoring of sedentary behavior [3].

On the other hand, the COVID-19 pandemic stimulated the development of telemedicine applications because all health systems had to face changes. Thus, remote patient–doctor communication was developed with the adjustment of treatment schemes and monitoring of symptoms, a better understanding on the part of patients of risk factors and the importance of cardiovascular prevention, as well as the motivation of patients to self-monitor and engage in self-care [18,19,20,21].

Although the frequency of face-to-face visits for blood pressure monitoring has decreased, studies have shown that many patients have enrolled in various telemedicine programs for remote blood pressure monitoring and treatment adjustments at home [20]. Strict government health recommendations to limit the spread of COVID-19 were followed by a reluctance of patients to go to the hospital and have led to a marked reduction in consultations and admissions for cardiovascular disease [22]. 

As in other studies, we found that despite the healthcare delivery barriers created by the COVID-19 pandemic, the use of telemedicine allowed us to not only continue taking care of patients with high cardiovascular risk scores but also to expand the monitoring far beyond the geographical boundaries of our previous catchment area. This is the first study analyzing the impact of telemedicine on cardiovascular counseling and testing during the COVID-19 pandemic in Romania. Our results support the ongoing use of telemedicine as a means to improve patient access to cardiovascular counseling and testing services [10,21,22,23,24].

All parameters of the lipogram (with the exception of HDL cholesterol) had an increasing trend during the pandemic at the beginning but, after that, by enrolling patients in the telemedicine application, we managed to reduce it, especially in patients in the very high-risk group. Conversely, for HDL, we found a significant decrease in HDL levels in all risk groups during Lock and Restr-P, but, with telemedicine help, during the Rel-P period, its average level increased and even exceeded the pre-pandemic level. 

Taking into account the lipid-lowering medication, the telemedicine application helped us to adapt the statin, ezetimibe, and fibrate doses according to the lipidogram level, without the need for a face-to-face consultation. 

Thus, the secondary remote prevention measures for those at very high risk gave very good results, i.e., their cardiovascular risk score was kept relatively constant [25,26,27]. In addition, patients with CAD are used to living a healthier lifestyle, maintaining their lipid profile and blood sugar within the limits corresponding to the risk group. Therefore, the blockages did not impose a significant change in their daily life, except regarding the level of physical activity.

Furthermore, by significantly increasing the number of teleconsultations during the pandemic, we managed hypertension and patients’ symptoms without the need for exposure to the hospital environment. Despite the significant reduction in face-to-face consultations, there were no increases in hospitalizations and ED visits due to decompensated angina and no increase in cardiovascular mortality in our sample [28]. This reinforces the fact that this approach was safe and effective, and simultaneously allowed the protection of patients from exposure to COVID-19 [29].

On the other hand, the primary remote prevention measures, especially for those at high risk, did not have the expected results. An explanation could be the fact that this group of patients, being mostly asymptomatic and without cardiovascular antecedents, neglected themselves and failed to maintain a healthy lifestyle. In addition, many of these patients were diabetic and blood sugar was the only laboratory parameter that increased significantly during the pandemic and remained elevated even after the pandemic in the studied group [18].

Moreover, blood sugar was high in all risk groups, but especially in those with medium-low risk. An explanation could be the fact that among them the percentage of anti-COVID-19 vaccinated people was lower and, as a consequence, they had more frequent moderate–severe forms of infection with COVID-19. After infection with COVID-19, many of the patients developed diabetes, with the percentage of diabetic patients in Rel-P being 10.76% compared to 6.3% in the pre-pandemic period. In addition, there was a good correlation between infection with COVID-19 and newly discovered DM after the pandemic.

On the other hand, with the exception of blood sugar, all the parameters monitored in our study returned to the pre-pandemic level or very close to it in the second year of the pandemic. The percentage of obese and smokers increased during the pandemic, but through primary and secondary prevention measures using the telemedicine application, we managed to reduce it. Interestingly, although physical activity decreased significantly in the first year of the pandemic, in Rel-P, people became more active than before the pandemic. One explanation for this is that the remote monitoring program, which included patient education, mitigated the impact of the isolation measures, allowing the continuous monitoring of vital parameters and ensuring counseling and the timely adjustment of a therapeutic regimen. Thus, by keeping patients in contact with the medical team (through phone calls and online consultations) and offering opportunities to improve their lifestyle and reduce cardiovascular risk, towards the end of the pandemic, the patients enrolled in the study became more active and improved their eating habits. Additionally, through all digital means, our clinic aggressively promoted physical activity for the whole family, adhering to a healthy diet, and reducing caloric intake to avoid weight gain [28,29,30,31,32]. Despite the physical distance, we tried to interact with patients virtually, including group fitness classes, sports for seniors, running on the treadmill, and stationary cycling in some online sessions. Furthermore, in compliance with social distancing rules, we encouraged patients to exercise.

Looking at these positive preliminary results, our study, like others, encourages the incremental use of telemedicine for cardiovascular prevention [29,33,34,35]. It is a feasible, safe, and efficient method to ensure access to the health system which is frequently overloaded. From this perspective, this approach can be extended in situations where physical consultation is not possible for logistical reasons, such as patients with reduced mobility or who live at great distances.

Thus, although our model is based on the experience of a private clinic of cardiovascular diseases, by expanding it and addressing all these issues, including those related to interoperability, all barriers can be overcome and can facilitate the implementation of safe and effective virtual primary and secondary prevention [34,35,36,37,38,39,40,41]. Finally, future studies must evaluate the cost, effectiveness, and sustainability of these models as well as the workload of physicians. Thus, care and prevention models through telemedicine, based on multidisciplinary teams and using interoperable applications at the national level, have the potential to increase their medical value and decrease doctors’ workloads.

### 4.1. Limitations of the Study

There are some limitations to our study: it is a single-center study and had a short period of follow-up during the pandemic compared with our pre-pandemic period and a low event number. However, we evaluated a large number of parameters. Unlike other monitoring strategies that rely on direct-to-consumer technologies (e.g., smartphones), which are more likely to appeal to younger patient populations, the system used in this study was provided and supported by our clinic. We could not exclude that some patients already applied for some form of self-quarantine. Because the present findings were obtained with a multiparametric approach in a structured remote monitoring center, they represent a complex intervention, and their generalizability to different technologies or other organizations is unknown. 

Our study also highlighted some of the limitations of utilizing telemedicine. During an in-person patient encounter, obtaining a patient blood sample is very easy. However, 14% of patients failed to go to the testing laboratory. In addition, we were unable to perform physical examinations via telemedicine. That is why another difficulty was the fact that several older patients preferred to defer telemedicine visits in favor of obtaining in-person visits, despite their higher risk for COVID-19 exposure. This may have been due to a lower comfort level with telemedicine technology in older adults. 

On the other hand, because of the interoperability problems encountered in our country with patients’ medical records, the electronic registries were not systematized and there is no software that can generate an alert email every time one of the CVD patients was admitted to the hospital.

Moreover, our study did not address the satisfaction level of patients or clinicians related to the dedicated telemedicine application, which is an important aspect that should be addressed in future qualitative studies. Additionally, we did not have information regarding specific technical difficulties during telemedicine visits.

The results of our study are based on the information obtained at a certain moment of the health crisis and do not represent the general experience in the practice of cardiovascular prevention. Further research could evaluate the perspectives regarding cardiovascular online prevention and the implications of its use on a large scale and for a more sustained period and in a non-pandemic context.

### 4.2. Perspectives

The COVID-19 pandemic and all its associated limitations have forced the healthcare system to rapidly adopt remote monitoring strategies. Until recently, the difficulties encountered and the lack of consistent benefits observed in randomized trials of remote monitoring applications in patients with CAD have prevented the widespread adoption of this approach. Moreover, primary prevention took second place during the pandemic.

Our application is still under evaluation, but the COVID-19 crisis created unique opportunities to examine the potential of remote prevention. Our study suggests that a combination of remote monitoring with less frequent face-to-face visits is a good method for primary and secondary prevention while maintaining the safety of CAD patients. In summary, our experience highlights the benefits of this remote prevention strategy in various risk groups of patients and encourages larger studies to confirm our conclusions.

## 5. Conclusions

Lipid levels increased significantly during the pandemic in the first year, but after that, we managed to lower them, especially in those with a very high risk for CVD.

The blood glucose level increased in all risk groups, but especially in the medium-low risk group, probably also because, being less vaccinated, they developed moderate–severe forms of COVID-19 and many remained diabetic after the infection.

The cardiovascular risk score showed an increasing trend during the Restr-P but by using the telemedicine application and an increase in medication we managed to reduce it. However, most of the scores remained higher than the pre-pandemic level. Physical inactivity was an exception to this rule: the percentage of inactive people increased during the pandemic but significantly reduced at the end of the pandemic compared to the pre-pandemic period.

The telemedicine application for primary and secondary cardiovascular prevention was good, especially for secondary prevention in very high-risk groups, and during the second year, after the learning curve was overcome, the patients were more receptive and the stress generated by the pandemic was reduced.

## Figures and Tables

**Table 1:** Baseline characteristics of the study groups.

Variables	TOTAL pts N = 3439	Group 1—194 pts Very High Risk	Group 2—1611 pts High Risk	Group 3—1634 pts Medium and Low Risk	*p*-Value (ANOVA, Pearson Chi-Square)
Mean (SD) age (years)	61.54 (16.26)	72.01 (11.07)	64.89 (14.19)	57.00 (17.29)	0.0000001
Women, no. (%)	1905 (55.4%)	42 (21.6%)	807 (50.1%)	1056 (64.6%)	0.0000001
Medical history, no. (%)					
Arterial hypertension	852/1554 (54.8%)	120/187 (64.2%)	352/585 (60.2%)	380/782 (48.6%)	0.000003
Diabetes mellitus	217 (6.3%)	49 (25.3%)	168 (10.4%)	0 (0.0%)	0.0000001
Paroxysmal atrial fibrillation	765/3439 (22.2%)	65/194 (33.5%)	418/1611 (25.9%)	282/1634 (17.3%)	0.0000001
Heart failure	98/1555 (6.3%)	13/188 (6.9%)	47/585 (8.0%)	38/782 (4.9%)	0.053710
Obesity (BMI > 30)	470/1494 (31.5%)	54/180 (30.0%)	213/584 (36.5%)	203/730 (27.8%)	0.003186
Smokers	221/1508 (14.7%)	20/182 (11.0%)	129/584 (22.1%)	72/742 (9.7%)	0.0000001
Sedentary	539/1508 (35.7%)	65/182 (35.7%)	220/584 (37.7%)	254/742 (34.2%)	0.431005
Stress	1083/1508 (71.8%)	117/182 (64.3%)	495/584 (84.8%)	471/742 (63.5%)	0.0000001
Alcohol consumption	507/1508 (33.6%)	53/182 (29.1%)	261/584 (44.7%)	193/742 (26.0%)	0.0000001
Mean (SD) initial cardiovascular risk score	5.16 (2.106)	5.96 (2.027)	6.87 (1.365)	3.59 (1.216)	0.0000001
Mean (SD) heart rate	72.09 (11.861)	65 (11.546)	70 (13.673)	75 (14.563)	0.0000001
Mean (SD) systolic blood pressure (mm Hg)	138.59 (21.535)	143.39 (19.581)	140.76 (20.868)	135.82 (22.101)	0.0000001
Mean (SD) diastolic blood pressure (mm Hg)	78.81 (11.651)	77.89 (8.917)	78.84 (11.657)	79.02 (12.212)	0.490247

BMI—body mass index.

**Table 2:** Dyslipidemia treatment in the study groups.

Dyslipidemia Initial Treatment, no. (%)	Total pts N = 3439	Group 1—194 pts Very High Risk	Group—1611 pts High Risk	Group 3—1634 pts Medium and Low Risk	*p*-Value (Pearson Chi-Square)
Statins, no. (%)—total	1619 (47.1%)	181 (93.3%)	821 (51.0%)	617 (37.8%)	0.000001
Rosuvastatin	668 (41.3%)	72 (39.8%)	374 (45.6%)	222 (36.0%)	
Atorvastatin	866 (53.5%)	104 (57.5%)	397 (48.4%)	365 (59.2%)	
Simvastatin	83 (5.1%)	5 (2.8%)	48 (5.8%)	30 (4.9%)	
Other statins	2 (0.2)	0 (0.0%)	2 (0.2%)	0 (0.0%)	
Fenofibrat	237 (6.9%)	33 (17.0%)	139 (8.6%)	65 (4.0%)	0.000001
Ezetimib	196 (5.7%)	16 (8.2%)	107 (6.6%)	73 (4.5%)	0.008151
Omega 3	753 (21.9%)	59 (30.4%)	349 (21.7%)	345 (21.1%)	0.016274
Combination (statins + ezetimib)	206 (6.0%)	37 (19.1%)	126 (7.8%)	43 (2.6%)	0.000001

**Table 3:** The cardiovascular risk score and modifiable cardiovascular risk factors during the pandemic.

Variables	Pre-Pandemic (Pre-P) 1 March 2019–1 March 2020	Restricted-Pandemic Lockdown (Lock) 1 March 2020–1 September 2020	Restricted-Pandemic (Restr-P) 1 September 2020–1 March 2021	Relaxed-Pandemic (Rel-P) 1 March 2021–1 March 2022	*p*-Value (ANOVA, Pearson Chi-Square)
Cardiovascular risk score	5.16 ± 2.105	5.56 ± 2.005	5.77 ± 1.835	5.83 ± 1.846	0.000001
Arterial hypertension, no. (%) (systolic blood pressure > 140 mmHg or diastolic blood pressure > 90 mmHg)	1891 (54.9%)	2137 (62.1%)	2297 (66.79%)	1992 (57.92%)	0.000001
Diabetes mellitus, no. (%)	217 (6.3%)	267 (7.76%)	334 (9.71%)	370 (10.76%)	0.000003
Smokers, no. (%)	515/3439 (14.97%)	551 (16.02%)	566 (16.46%)	529 (15.38%)	0.04127
BMI	28.92 ± 19.525	29.61 ± 12.614	30.33 ± 8.751	28.36 ± 7.810	0.041
Obesity (BMI > 30), no. (%)	1083 (31.49%)	1267 (36.84%)	1387 (40.33%)	1121 (32.59%)	0.003157
Physical inactivity, no. (%)	539 (15.7%)	701 (20.38%)	810 (23.55%)	334 (9.7%)	0.000003
Unhealthy diet, no. (%)	1046 (30.41%)	1230 (35.76%)	1310 (38.09%)	1002 (29.13%)	0.003157
Mean systolic blood pressure (mmHg)	138.59 ± 21.535	147.25 ± 21.278	147.87 ± 21.091	138.97 ± 21.562	0.000001
Mean diastolic blood pressure (mmHg)	78.81 ± 11.651	79.881 ± 12.013	80.23 ± 11.402	78.13 ± 11.142	0.000001
Mean HR (b/min)	N = 1555 72.09 ± 11.861	N = 1108 73.44 ± 12.053486	N = 1314 72.96 ± 11.916	N = 959 72.08 ± 12.547	0.051000
Heart failure, no, (%)	98/1555 (2.8%)	80/1108 (2.3%)	108/1314 (3.1%)	87/959 (2.5%)	0.05136

HR—heart rate; BMI—body mass index.

## Data Availability

All data generated or analyzed during this study are included in this published article.

## Supplementary Materials

The following supporting information can be downloaded at: https://www.mdpi.com/article/10.3390/healthcare11121727/s1, Table S1. Comparison of total cholesterol values between study periods. Table S2. Comparison of LDL-cholesterol values between study periods. Table S3. Comparison of HDL-cholesterol values between study periods. Table S4. Comparison of TGL values between study periods. Table S5. Comparison of mean glucose levels between study periods. Table S6. Comparison of uric acid values between study periods. Figure S1. Study flow-chart.
