# Supplementary material for: Challenges in Caring for People with Cardiovascular Disease through and beyond the COVID-19 Pandemic: The Advantages of Universal Access to Home Telemonitoring
Source: Healthcare (Basel). 2023 Jun 12;11(12):1727. doi: 10.3390/healthcare11121727 (PMC10298744; doi:10.3390/healthcare11121727)
Supplement: Supplementary file 1 [file healthcare-11-01727-s001.zip › healthcare-2342945-supplementary.pdf]

Table S1. Comparison of total cholesterol values between study periods.

Total cholesterol (mg/dl)	Lock<br>1 march 2020 – 1 sept 2020	Restr-P<br>1 sept 2020 - 1 march 2021	Rel-P<br>1 march 2021 - 1 march 2022
Pre-P	Pre-P: 183.05± 48.196<br>Lock: 216.72± 54.194<br>N=1703<br>**P_value= 0.000001**	Pre-P: 181.98± 47.965<br>Restr-P: 218.98± 59.211<br>N=1849<br>**P_value= 0.000001**	Pre-P: 187.06± 47.239<br>Rel-P: 180.04± 46.030<br>N=1332<br>**P_value= 0.000001**
Lock	X	Lock: 221.93± 54.487<br>Restr-P: 226.79± 61.673<br>N=1327<br>**P_value= 0.000001**	Lock: 223.35± 54.310<br>Rel-P: 180.23± 46.139<br>N=1254<br>**P_value= 0.000001**
Restr-P	X	X	Restr-P: 227.82± 61.458<br>Rel-P: 180.00± 46.167<br>N=1303<br>**P_value= 0.000001**

Pre-P- prepandemic; Restr-P- restrictive pandemic; Rel-P- relaxed pandemic; Lock- lockdown.

Table S2. Comparison of LDL-cholesterol values between study periods.

LDL cholesterol mg/dl	Lock<br>1 march 2020 – 1 sept 2020	Restr-P<br>1 sept 2020 - 1 march 2021	Rel-P<br>1 march 2021 - 1 march 2022
Pre-P	Pre-P: 113.21± 41.046<br>Lock: 136.75± 43.602<br>N=1557<br>**P_value= 0.000001**	Pre-P: 112.18± 40.582<br>Restr-P: 137.57± 45.620<br>N=1649<br>**P_value= 0.000001**	Pre-P: 116.55± 40.145<br>Rel-P: 112.39± 40.470<br>N=1278<br>**P_value= 0.000001**
Lock	X	Lock: 136.65± 45.289<br>Restr-P: 138.82± 47.991<br>N=1263<br>**P_value= 0.001519**	Lock: 138.36± 44.411<br>Rel-P: 112.25± 40.969<br>N=1216<br>**P_value= 0.000001**
Restr-P	X	X	Restr-P: 140.17± 47.752<br>Rel-P: 111.50± 40.640<br>N=1244<br>**P_value= 0.000001**

LDL- low density lipoprotein; Pre-P- prepandemic; Restr-P- restrictive pandemic; Rel-P- relaxed pandemic; Lock- lockdown.

Table S3. Comparison of HDL-cholesterol values between study periods.

**HDL-cholesterol (mg/dl)**	**Lock 1 march 2020 – 1 sept 2020**	**Restr-P 1 sept 2020 - 1 march 2021**	**Rel-P 1 march 2021 - 1 march 2022**
Pre-P	Pre-P: 52.58± 9.911<br>Lock: 47.90± 13.815<br>N=1567<br>**P_value= 0.000001**	Pre-P: 52.89± 9.988<br>Restr-P: 45.23± 13.829<br>N=1675<br>**P_value= 0.000001**	Pre-P: 50.97± 10.188<br>Rel-P: 57.81± 14.620<br>N=1347<br>**P_value= 0.000001**
Lock	X	Lock: 47.63± 15.969<br>Restr-P: 43.31± 14.027<br>N=1243<br>**P_value= 0.000001**	Lock: 47.38± 16.448<br>Rel-P: 57.28± 14.153<br>N=1219<br>**P_value= 0.000001**
Restr-P	X	X	Restr-P: 42.56± 13.967<br>Rel-P: 58.11± 15.000<br>N=1295<br>**P_value= 0.000001**

HDL- high density lipoprotein; Pre-P- prepandemic; Restr-P- restrictive pandemic; Rel-P- relaxed pandemic; Lock- lockdown.

Table S4. Comparison of TGL values between study periods.

**TGL (mg/dl)**	**Lock 1 march 2020 – 1 sept 2020**	**Restr-P 1 sept 2020 - 1 march 2021**	**Rel-P 1 march 2021 - 1 march 2022**
Pre-P	Pre-P: 121.83± 76.590<br>Lock: 158.29± 68.000<br>N=1666<br>**P_value= 0.000001**	Pre-P: 120.10± 68.755<br>Restr-P: 174.72± 64.205<br>N=1795<br>**P_value= 0.000001**	Pre-P: 124.70± 67.988<br>Rel-P: 126.46± 59.727<br>N=1321<br>P_value= 0.122975
Lock	X	Lock: 159.22± 64.172<br>Restr-P: 177.37± 63.033<br>N=1300<br>**P_value= 0.000001**	Lock: 160.12± 64.954<br>Rel-P: 125.75± 60.144<br>N=1243<br>**P_value= 0.000001**
Restr-P	X	X	Restr-P: 177.96± 63.577<br>Rel-P: 126.38± 60.159<br>N=1284<br>**P_value= 0.000001**

TGL- triglycerides; Pre-P- prepandemic; Restr-P- restrictive pandemic; Rel-P- relaxed pandemic; Lock- lockdown.

Table S5. Comparison of mean glucose levels between study periods.

Glucose (mg/dl)	Lock<br>1 march 2020 – 1 sept 2020	Restr-P<br>1 sept 2020 - 1 march 2021	Rel-P<br>1 march 2021 - 1 march 2022
Pre-P	Pre-P: 104.23± 28.326<br>Lock: 117.11± 28.738<br>N=1641<br>**P_value= 0.000001**	Pre-P: 103.26± 28.161<br>Restr-P: 119.68± 31.487<br>N=1760<br>**P_value= 0.000001**	Pre-P: 105.10± 25.720<br>Rel-P: 137.18± 32.819<br>N=1273<br>**P_value= 0.000001**
Lock	X	Lock: 118.78± 27.255<br>Restr-P: 123.76± 30.192<br>N=1294<br>**P_value= 0.000001**	Lock: 119.32± 26.630<br>Rel-P: 138.40± 32.212<br>N=1203<br>**P_value= 0.000001**
Restr-P	X	X	Restr-P: 123.99± 29.271<br>Rel-P: 137.16± 32.864<br>N=1268<br>**P_value= 0.000001**

Pre-P- prepandemic; Restr-P- restrictive pandemic; Rel-P- relaxed pandemic; Lock- lockdown.

Table S6. Comparison of uric acid values between study periods.

Uric Acid (mg/dl)	Lock<br>1 march 2020 – 1 sept 2020	Restr-P<br>1 sept 2020 - 1 march 2021	Rel-P<br>1 march 2021 - 1 march 2022
Pre-P	Pre-P: 5.26± 1.554<br>Lock: 6.12± 2.716<br>N=1430<br>**P_value= 0.000001**	Pre-P: 5.29± 4.139<br>Restr-P: 6.18± 1.797<br>N=1513<br>**P_value= 0.000001**	Pre-P: 5.39± 1.526<br>Rel-P: 5.80± 2.919<br>N=1142<br>**P_value= 0.000001**
Lock	X	Lock: 6.26± 2.947<br>Restr-P: 6.41± 1.818<br>N=1120<br>P_value= 0.072092	Lock: 6.25± 2.975<br>Rel-P: 5.79± 2.957<br>N=1093<br>**P_value= 0.000001**
Restr-P	X	X	Restr-P: 6.44± 1.809<br>Rel-P: 5.82± 2.975<br>N=1085<br>**P_value= 0.000001**

Pre-P- prepandemic; Restr-P- restrictive pandemic; Rel-P- relaxed pandemic; Lock- lockdown.

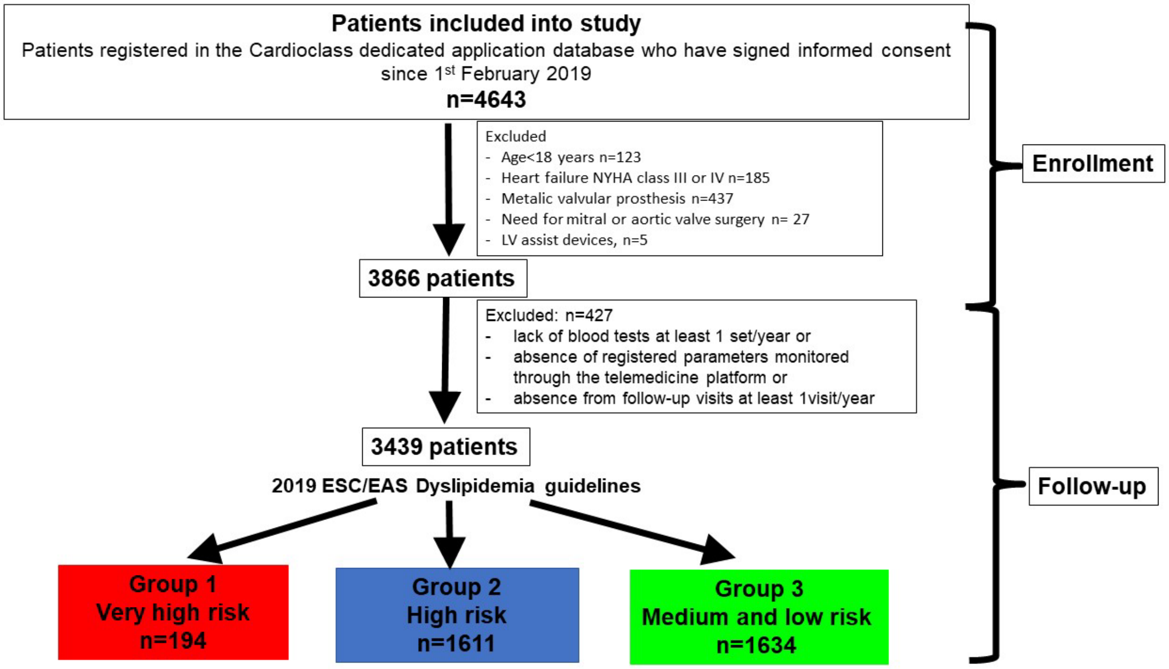

Figure S1. Study flow-chart.
